# Community-Engaged Scholarship: An Interpartner Approach for Collaborative Practice

**DOI:** 10.1007/s42822-025-00239-z

**Published:** 2026-01-29

**Authors:** Trina D. Spencer, Valerie Thompson, Jomella Watson-Thompson

**Affiliations:** Department of Applied Behavioral Science, University of Kansas, 4001 Dole Human Development Center, 1000 Sunnyside Avenue, Lawrence, KS 66045 USA

**Keywords:** Interprofessional collaboration, Interdisciplinary, Community-engaged scholarship, Collaboration, Training

## Abstract

The need to prepare future behavior analysts to become effective, collaborative professionals is increasingly evident. The social importance of goals, procedures, and effects has always been a critical part of our applied science; yet too often it is superseded in priority by considerations of procedural integrity. Disciplinary centrism, often cultivated through traditional training models, can be counterbalanced through intentional promotion of cultural and professional humility. We argue that community-engaged scholarship (CES) offers a behaviorally compatible framework for collaboration training and emphasizes the same collaboration skills should extend beyond interprofessional collaborations to include nonprofessionals (e.g., clients, families, communities). Because CES emphasizes reciprocal learning “about, from, and with” all potential partners—including those receiving services—and naturally positions students alongside nonbehavioral collaborators with varied lived experiences and professional perspectives, we use the term interpartner as an alternative to interprofessional. When CES is paired with the Interprofessional Education and Collaborative Practice (IPECP) competencies—values and ethics, roles and responsibilities, interprofessional communication, and teams and teamwork—it creates a powerful, inclusive model for cultivating collaboration skills in behavior analytic trainees. This blended approach aligns with the core aims of applied behavior analysis while advancing its relevance and responsiveness in diverse service contexts. In this paper, we first provide an overview of key concepts from IPECP and CES. We then illustrate the application of the Participatory Action Cycle for Community Engagement through two case examples that highlight how CES can serve as a foundational context for interpartner education within behavior analytic training.

In recent years, opposition to behavior analytic research and practice has been noted (Anderson, 2023; Kirkham, 2017; Sandoval-Norton & Shkedy, 2019). While behavior analysts often engage in positive and meaningful interactions with individuals, families, and other professionals, there have been some concerns raised about collaborative skills (Henderson et al., 2023; Slim & Reuter-Yuill, 2021). Although such critiques can be minimized or dismissed, a more productive and alternative response involves listening carefully, engaging in self-reflection, communicating with clarity, and seeking common ground (Kirby et al., 2022).

While unpacking the heart of our science, Wolf (1978) proposed that social validity should be elevated to the same status as objective measurement if applied behavior analysis is to meaningfully address problems of social importance. On the basis of Wolf’s humble reflection of his own experiences, he noted a systematic disconnect between procedural effectiveness and social relevance across the discipline and concluded that society is best qualified to determine what is socially important. Collaborative practice can help center social validity in behavior analytic practice and research with attention to the social importance of the goals, procedures, and effects (Wolf, 1978).

When well-intentioned services are perceived as unacceptable or harmful, this may signal a failure to establish or maintain social validity. Operating in a vacuum, without feedback, stakeholder input, or peer consultation may reduce the likelihood of contacting the discriminative stimuli and reinforcement contingencies that shape and maintain ethical and socially valid practice. Over time, the absence of collaborative feedback can contribute to response drift, thereby allowing practice to deviate, unknowingly, from acceptable and socially valid norms. Collaborative engagement provides the cues, consequences, and opportunities for integrated feedback that is necessary to maintain alignment with both evidence-based practice and the evolving values of the communities served. Yet, until there is a collective commitment to address the barriers that prevent such engagement, there remains a risk that behavior analytic practice will continue perpetuating the longstanding patterns of disconnect between procedural effectiveness and social relevance, undermining our impact and credibility.

The *Behavior and Social Issues* special topic on interprofessional collaboration may signal growing interest in this area. However, there remains a significant need for clear guidance on how behavior analysts can contribute effectively as valued collaborators. This paper proposes a potential roadmap, acknowledging that the content is based on broad generalizations and approximations, which further underscores the need for ongoing refinement through emerging evidence and continued social validation of our services. Although reflecting critically on our past mishaps may be uncomfortable, it is a necessary condition for meaningful progress. Grounded in our foundational principles, we envision a future in which behavior analysts are sought out not only for their technical expertise, but for their capacity to collaborate with professional humility and socially valid effectiveness.

## From Disciplinary Centrism to Collaboration: The Evolution of Applied Behavior Analysis

Conditioned by disciplinary training practices, some behavior analysts may have more limited skills and experience in collaborating across fields, which may be rooted in disciplinary centrism—the belief that professionals from one’s own field are better trained or more knowledgeable than those from other disciplines (Kirby et al., 2022; Pecukonis, 2020). During its early development, behavior analysis needed to differentiate itself from psychology to establish a distinct disciplinary identity, a move that helped justify discipline-centric training models. This emphasis on disciplinary rigor may have contributed to limited norms around cross-disciplinary collaboration and professional humility. In practice, effective collaboration requires more than technical competence; it also necessitates the ability to recognize and value the expertise of others, a skill that may be underdeveloped among behavior analysts due to limited formal training and experience in cross-disciplinary engagement (Boivin et al., 2021; Kelly & Tincani, 2013; Kirby et al., 2022).

In applied behavior analysis, there are mounting calls to listen to and take seriously the criticisms voiced by clients and colleagues, as this feedback is essential for the continued evolution of our field (Friedman et al., 2024; Kirby et al., 2022). As we reflect on the emergence of our applied science and the potential it holds to positively impact society (Baer et al., 1968), we remain committed to that original aim. To help move the field forward (even incrementally), as such, a model is proposed for training behavior analysts to become successful collaborators, accompanied by two case studies that illustrate its application.

## Collaborative Practice

To determine *how* we achieve our aims, we must first clarify *what* is to be accomplished and *why*. An important goal for our field, particularly for university faculty involved in training future behavior analysts, is to design learning environments that effectively cultivate the behavioral repertoires essential for successful collaborative practice (Boivin et al., 2021; Friedman et al., 2024). Collaborative practice involves professionals from diverse disciplines and backgrounds working together with each other, with clients, and with families and communities to provide the highest quality of care (World Health Organization [WHO], 2010). While additional research is needed, existing evidence suggests that collaborative practice improves outcomes more effectively than single-discipline service delivery models (Reeves et al., 2017). Additional benefits include improved communication and coordination across team members and more holistic treatment that addresses multiple facets of a client’s life (Lee et al., 2021; Reeves et al., 2013). To advance collaborative practice, we should consider its complexity and varying forms.

### Understanding Collaboration: Disciplinary and Multisector Collaboration

Collaboration is not a singular concept but rather exists along a continuum of engagement. Himmelman (2002) outlines levels of collaborative relationships, ranging from structured networking and coordination to cooperation and full collaboration. These levels progress from basic information exchange to sharing resources, integrating activities, and building capacity toward a shared goal. As engagement deepens along this continuum, collaborators increasingly share risks, resources, responsibilities, and rewards.

Disciplinary collaboration also occurs along a spectrum, from multidisciplinary approaches, where professionals contribute expertise within their own domains, to interdisciplinary work, which involves coordinated efforts and shared goals, and ultimately to transdisciplinary collaboration. The transdisciplinary model represents the highest level of integration, in which roles expand, expertise is blended, and problem-solving is shared across disciplines (Bowman et al., 2021; D’Amour et al., 2005; Slim & Reuter-Yuill, 2021). Kirby et al. (2022) argued that collaborating effectively with professional colleagues from other disciplines is necessary to scale our services and have the broadest impact possible.

Another important lens for understanding collaborative practice is multisector engagement, which involves coordinated action across different community sectors, systems, or areas of the community. Through multisector collaborations, stakeholders from systems such as education, healthcare, business, and law enforcement, who influence or are impacted by an issue, work together toward a common goal (Fawcett et al., 2010). These partnerships integrate resources and implement multiple strategies (i.e., interventions) across socioecological levels (e.g., client, family, community) to collectively address shared goals, often necessary to address complex, systemic issues (Watson-Thompson et al., 2008). Multisector collaborations include a range of stakeholders, including professionals, residents, advocates, and academicians, who may contribute complementary knowledge, lived experience, and enhance the collective capacity of the effort. For example, in adolescent substance abuse prevention efforts, 12 key community sectors are typically engaged in collaborative action. The involved sectors include youth, parents, schools, youth-serving organizations, businesses, media, law enforcement, religious or fraternal organizations, civic groups/volunteers, healthcare, state, local or tribal government, and other community organizations. Partners across these sectors often convene through coalitions to support shared goals. For instance, schools, businesses, parents, and law enforcement may collaborate through a coalition to prevent underage drinking by implementing and enforcing policies that restrict illegal alcohol sales and distribution to youth at home or community events.

## Interprofessional Collaboration: An Outcome of Interprofessional Education

Interprofessional education (IPE) occurs “when two or more professionals learn about, from, and with each other to enable effective collaboration and improve health outcomes” (WHO, 2010, p. 13). Interprofessional collaboration (IPC) is often considered a product or outcome of IPE, as it extends from IPE and emphasizes collaborative practice among professionals from different disciplines, supported by co-learning experiences and shared goals in service delivery, and client-centered care (Ruebling et al., 2014; WHO, 2010). To better define the types of activities that support Interprofessional Education and Collaborative Practice (IPECP), collaborative work is characterized as interprofessional teamwork, interprofessional collaboration, interprofessional coordination, and networks (Himmelman, 2002; Interprofessional Education Collaborative [IPEC], 2023). While IPE and IPC have often been used interchangeably, leading to some conceptual ambiguity, established domains and competencies help clarify the distinct skills and activities needed to advance both education and practice (IPEC, 2023).

On the basis of the Framework for Action on IPECP, there are well-established competencies that guide the implementation of IPC and IPE. Slim and Reuter-Yuill (2021) offered a behavioral interpretation of the Interprofessional Education Collaborative’s (IPEC; 2023) four core competencies for collaborative practice, which are briefly summarized. The first competency, Values and Ethics, emphasizes shared values, ethical practice, and mutual respect among team members. The second, Roles and Responsibilities, involves clarifying each team member’s knowledge and expertise to align it with the team’s collective goal of improving client outcomes. Interprofessional Communication, the third competency, highlights the importance of responsible, responsive, and respectful communication within the team. Finally, Teams and Teamwork focuses on applying principles of team science, allowing for flexible role adaptation to meet the evolving needs of the team and the individuals they serve.

### Interprofessional Education in Applied Behavior Analysis

Training behavior analysts alongside other professionals remains uncommon in our field. Kelly and Tincani (2013) examined the extent of IPE and collaboration training received by behavior analysts, as well as their attitudes toward collaboration. Their findings revealed that although behavior analysts frequently interact with a range of professionals and stakeholders, most received little to no formal training in collaboration. The survey also indicated that behavior analysts were more inclined to adopt recommendations from fellow behavior analysts than from interdisciplinary colleagues. Moreover, respondents expressed varied beliefs about the value of collaboration, some viewing it as necessary only when a problem cannot be solved independently. Kelly and Tincani (2013) concluded that additional training is essential to help behavior analysts develop effective collaboration skills. Since this research, there has been expanded interest in IPC and IPE for behavior analysts, including those working in autism treatment (Bowman et al., 2021; Henderson et al., 2023) and mental health spaces (Harvey et al., 2025; Summers et al., 2022). Altogether, these authors recommend the establishment of IPE models for training behavior analysts while acknowledging the need to generate behavior analytic research to inform their development and implementation.

To establish evidence-based IPE models within behavior analysis, it is important to acknowledge that the primary emphasis of IPE remains on collaboration in education and practice among professionals, although collaborative practice may also include clients, families, and communities (WHO, 2010). This emphasis reflects IPE’s origins in preparing health professionals across disciplines to work together to improve patient care (IPEC, 2023). Similarly, approaches focused on multidisciplinary, interdisciplinary, and transdisciplinary collaboration also are primarily centered on professional-to-professional relationships. While these approaches may support engaging nonprofessional stakeholders, including clients and their families, professional collaboration is still the focus and pathway for decision-making, service delivery, and outcomes. There remains a critical gap if professionals, within and across disciplines, are trained to collaborate with each other, but not necessarily with (not for) those they serve as equal and collaborative stakeholders.

Current IPE models emphasize learning with, from, and about other professionals, but may underrecognize the lived expertise of nonprofessional stakeholders such as clients, families, and community partners. Without intentional learning with also broader stakeholders, professionals may lack the full range of collaborative skills necessary to be responsive and adaptive when providing services to a range of populations and across different contexts. Additionally, minimizing the opportunities for meaningful engagement and feedback by those most affected, and often responsible, for longer-term maintenance of behavioral practices in the natural setting, may limit generalization and adoption. As IPECP is considered as a potential framework for adoption in applied behavior analysis, it is important to consider potential challenges or areas of adaptation that may be necessary for using the IPECP models to train future behavior analysts.

## Interpartner Education and Collaboration: Engaging Professionals and Nonprofessionals

In most service-delivery settings, such as schools, clinics, and homes, behavior analytic services may be a component of a broader team-based approach to support individuals and families (U.S. Department of Education, Office of Special Education and Rehabilitation, 2022; Virginia Department of Education, 2020). Collaboration within interdisciplinary teams is essential for delivering effective, coordinated care. A survey by Kelly and Tincani (2013) found that 62% of behavior analysts reported collaborating daily with professionals from other disciplines, underscoring the prevalence of interdisciplinary work. Ethically, collaboration with other professionals is also required under the Behavior Analyst Certification Board’s Ethics Code 2.10 (2020).

Effective collaboration, however, extends beyond professional colleagues to include clients, their families, and communities (Horbanczuk et al., 2024). Behavior analysts frequently work alongside those most affected by the behavior change interventions, including those receiving services and those responsible for implementing and maintaining change in the natural environment (e.g., caregivers, teachers). To prepare and program for generalization (from the start), collaboration training should build on interprofessional models and emphasize learning about, from, and with all potential partners, including those being served. This inclusive approach equips behavior analysts and other professionals to engage meaningfully and collaborate effectively across diverse service contexts.

With intentional design and thoughtful implementation, it is possible to develop collaborative repertoires that generalize across partners, settings, and service sectors. Given the broad applicability of the behavioral repertoires underlying collaborative practice, a conceptual approach that is supported in behavior analysis should not only include interprofessionals, but also nonprofessionals, including those served or most impacted. As such, the term *interpartner* is proposed as an alternative to interprofessional when considering how to train behavior analysts to educate and collaborate with other professionals and nonprofessionals. Potentially considering interpartner education and interpartner collaboration as an alternative may expand upon the traditional notion of interprofessional education and collaboration. The focus on partner in the terminology explicitly includes clients, families, community members, and others situated in applied contexts as legitimate and integral collaborative partners. Whereas interprofessional collaboration emphasizes cooperation among trained specialists, it may overlook or make less prominent the critical contributions, expertise, and insights of those most directly involved and impacted by the intervention. Interpartner collaboration, by contrast, names and normalizes a participatory approach in which individuals with lived experience, contextual expertise, and cultural knowledge are recognized and integrated as active contributors from the onset.

This reframing acknowledges that the long-term success of collaborative partnerships, including client-centered outcomes, depends on trust, accessibility, and alignment with local needs. It also centers on the concept of social validity—the social importance of the goals, procedures, and outcomes of behavior analytic practice (Wolf, 1978). Adopting an interpartner perspective can strengthen collaboration training by promoting a comprehensive model that integrates both education and practice. This approach encourages current and future practitioners to intentionally engage a full range of partners in advancing socially significant work. The willingness of partners, including those served, to be involved may serve as a form of social validation.

As the field of applied behavior analysis considers interprofessional education and collaboration, and we believe the time has come to do so, it should be adopted and adapted in a way that reflects our values and the applied context of our discipline based on our learning history. At this juncture, it is important that we consider our verbal behavior and recognize the response that is evoked. As such, we propose that interpartner collaboration may offer a more holistic and participatory approach by emphasizing the importance of collaboration with both professional and nonprofessional stakeholders through education and practice.

## Community-Engaged Scholarship: Advancing Interpartner Education and Collaboration

**Fig. 1:**
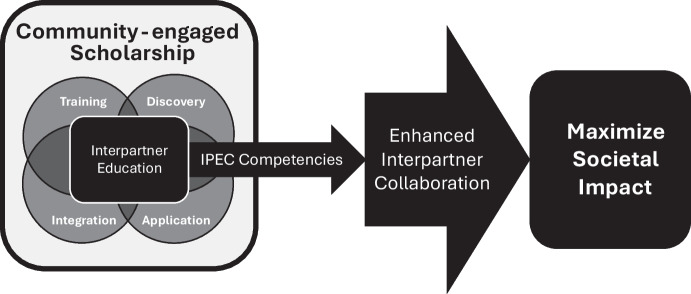
Theory of change for interpartner education and collaboration embedded in community-engaged scholarship

Given that much of our applied science and services stem from community engagement (Greenwood et al., 2025), community-engaged scholarship (CES) may offer a complementary approach that supports interpartner education and collaboration. By integrating interpartner education and collaboration approaches into community-engaged training and practice, applied behavior analysts may be better prepared to engage in participatory, socially valid, and meaningful collaborations through authentic partnership with those served in communities. Figure 1 outlines a theory of change and illustrates a proposed approach to interpartner education and collaboration, situated within the context of CES.

### Community-Engaged Scholarship

CES is grounded in the unification of training, discovery, integration, and application as interrelated functions that serve as vehicles for mutually beneficial collaboration (Boyer, 1996). These functions are not isolated but mutually reinforcing processes through which institutional (i.e., academic) and community-based actors co-create and apply knowledge to address socially significant concerns. Boyer’s model of CES emphasizes that research (discovery), synthesis across disciplines (integration), practical implementation (application), and education (teaching/training) should not be siloed. Instead, they function collectively as mechanisms that support mutually beneficial collaboration between scholars and communities.

Although CES approaches were developed within the context of academic-community partnerships, the principles may generalize to professionals in nonacademic settings. This integrated approach advances knowledge while addressing real-world challenges, ensuring that the work remains socially relevant and promotes participation from an array of partners. Broader adoption of CES in behavior-analytic training, research and practice may provide an approach for improving collaborative practice competencies.

Engagement is not solely defined by the location or content of applied work, but by the presence of reciprocal, trust-based processes that elevate the perspectives of all stakeholders throughout the life cycle of a project (Watson-Thompson & Thompson, 2023). It should be noted that although activities may be supported in an applied setting, it does not necessarily qualify it as engagement, which is a process by which there are mutually beneficial and reciprocal activities (Watson-Thompson & Thompson, 2023). Through meaningful engagement, social validity is programmed into the work, as stakeholders are continuously examining the social significance of the goals, procedures, and effects with those involved and impacted as a standard process. By combining teaching, discovery, integration, and application within a CES framework, and aligning these with IPEC competencies, professionals, including applied behavior analysts, are better positioned to contribute to collaborative efforts.

## CES Principles and IPEC Competencies

**Fig. 2:**
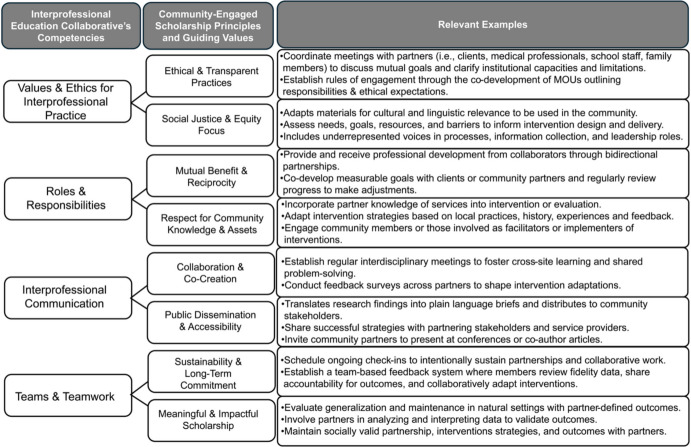
Alignment between interprofessional education collaborative (IPEC) competencies and community-engaged scholarship (CES) principles with examples

Figure 2 illustrates how the four core competencies identified by IPEC—values and ethics, roles and responsibilities, interprofessional communication, and teams and teamwork—align with long-standing principles of CES. Each pairing includes behavioral examples that show how these principles can be enacted in practice. This integrated model expands interprofessional education and collaboration by incorporating CES principles that support an interpartner approach to guide the development of meaningful, equitable, and sustainable partnerships, including with those served. Training behavior analysts in collaborative practice through interpartner approaches supports the development of IPEC competencies while also fostering professional humility and reducing disciplinary centrism—both essential for effective and inclusive collaboration (Kirby et al., 2022). Importantly, CES emphasizes the active involvement of those most impacted as equal collaborators in the process.

## Model for a Participatory Action Cycle for Community Engagement

**Fig. 3:**
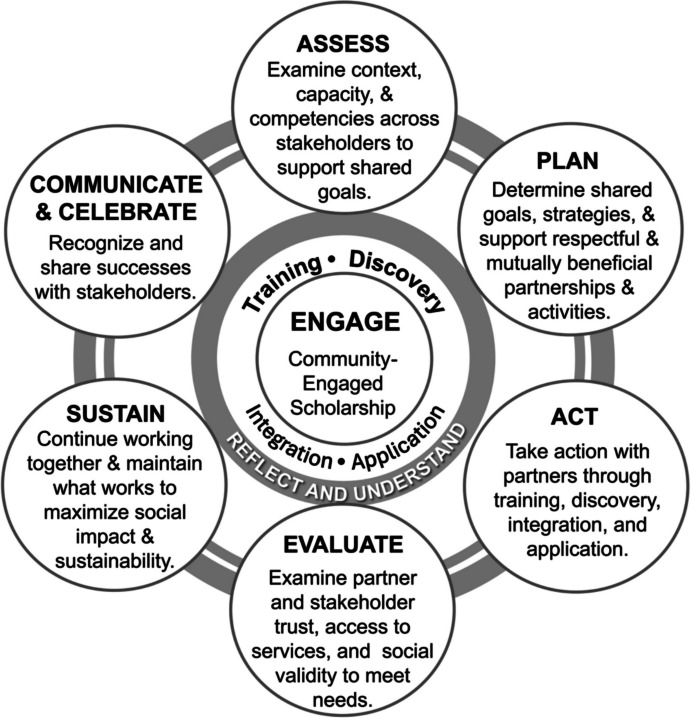
Participatory Action Cycle for Community Engagement. The integrated model demonstrates how community-engaged scholarship (CES) aligns with the components and key phases of the action cycle to provide guided recommendations for action. *Adapted from* “Action Cycle Model,” the Center for Community Health and Development, 2025, University of Kansas, https://ctb.ku.edu/en/get-started

Figure 3 presents a complementary framework grounded in CES, illustrating how interpartner education and collaboration can foster participatory action among a broad range of stakeholders, including both professionals and nonprofessionals. The Action Cycle model, developed by the Community Tool Box at the University of Kansas (Center for Community Health and Development, 2025), offers a structured, cyclical process with practical steps for advancing collaborative practices. This model aligns with CES principles and extends IPEC competencies by supporting meaningful, equitable, and sustained engagement, including with both professionals and nonprofessional stakeholders (e.g., clients, community). The Participatory Action Cycle for Community Engagement is an adapted model and provides recommendations for action to guide collaborative education and practice that supports an interpartner approach through CES principles and aligned IPEC competencies.

Each phase of the framework—Engage, Assess, Plan, Act, Evaluate, Sustain, and Communicate & Celebrate—reflects how community engagement can be leveraged to further extend the IPEC competencies and foster professional and cultural humility. The model helps to operationalize collaborative practices that can be supported through interpartner collaboration as an actionable, iterative process, continuously informed by engagement, applied learning, and reflective practice. By connecting the conceptual foundation (Fig. 1) to a strategic action cycle (Fig. 3), the model demonstrates that the path to maximizing societal impact is not linear. Rather, it is built through repeated, intentional engagement in applied contexts that supports integration, training, discovery, and application through the implementation of collaborative practices with those served whether through academic training, research, or practice. Interpartner education and collaboration offers behavior analysts a coherent and values-aligned roadmap for cultivating collaborative competence in ways that are sustainable, equitable, and socially meaningful with both professionals and nonprofessional stakeholders.

### Guidelines for Implementing the Participatory Action Cycle for Community Engagement

Using the Participatory Action Cycle for Community Engagement, we offer guidelines to help implement practices that support interpartner education and collaboration.

1. **Engage and Reflect:** Determine the context for engagement with the individual, group, and/or community impacted or served. Consider if training, discovery, integration, and/or application is the goal to be supported with the stakeholders involved in interpartner education or collaboration. Throughout the process, continuously reflect on how collaborators, including professionals and nonprofessional stakeholders, are being equitably involved and supporting meaningful partner engagement.
  - Clarify the purpose and goals for engagement with stakeholders.
  - Consider the array of engagement strategies and activities that can be supported across the stakeholders.
  - Identify those who should be engaged, including those most impacted. Determine how to make participation easier and more rewarding for all stakeholders.
  - Determine the function of those engaged and how individuals and groups involved will interact (e.g., roles, responsibilities, decision structure, ground rules).
  - Examine your own potential biases and be aware of how disciplinary centrism may influence opinions, attitudes, and behaviors for interacting with others. Develop and practice a posture of professional humility and cultural reciprocity involving self-reflection, listening, validating, and compromising (Kirby et al., 2022).
  - Ensure ongoing and systematic reflection of the CES and IPEC principles and practices for continuous and ongoing learning and adaptation.
2. **Assess:** Assess the institutional and community context, as well as the readiness and capacity of the individuals and groups interested or involved. Understand the issues that matter to the stakeholders, including through participation of both professionals and nonprofessionals, and the resources that are available. Examine the need or goal to be addressed by the stakeholders.
  - Examine the institutional and community capacity to support the goal. Consider both the community and institutional (e.g., university) context and conditions that may support or inhibit CES and interpartner education (Watson-Thompson, 2015). Proactively address potential challenges or barriers that may be within your context (e.g., course, degree program) and control (e.g., partner agreements).
  - Enhance stakeholder capacity. Seek to understand the history of engagement between the professional and nonprofessional partners, which may influence the CES and interpartner education activities. Learn from and with the client or community partners first and understand the needs and values of a partnership and those served from their perspective.
  - Determine and provide experiences that can enhance the competency and capacity of the collaborative stakeholders to identify and support the needs and goals. Ensure opportunities for co-learning and sharing across stakeholders, including uplifting the voice and perspective of those most affected.
  - Identify and gain consensus on the community-determined goals, needs and opportunities for engagement.
  - Inventory the resources, assets, experiences, and history available, including across stakeholders, and how the group can use them to support the shared goals (e.g., people, networks, skills, expertise).
3. **Plan:** Plan with partners in the community to support community engagement activities.
  - Engage proactively with potential or existing partners.
  - Ensure reciprocity and value in the activities to be supported. Determine what may be the long-term benefit and a sustainable approach that is focused on community-identified needs with the partners.
  - Identify the strategies to be supported to achieve the goals and determine those who can assist in implementing the activities. Be open to a variety of strategies that utilize the knowledge and expertise across stakeholders, including the client or community collaborators.
  - Ensure time to develop trust, rapport, and agreed upon guidelines for the expectations and commitment levels of the collaborative partners.
4. **Act:** Facilitate action to train and implement community-informed engagement strategies with partners in communities.
  - Establish practices, including courses and/or practicum experiences, to provide training in collaborative practices and to support opportunities to demonstrate competency with different partners and across settings.
  - Ensure training and support in implementing agreed upon strategies by stakeholders. Promote co-learning with and across professional and nonprofessional stakeholders recognizing all have value, knowledge, and experience.
  - As appropriate, implement the community-informed engagement strategies and interpartner education activities with partners in the community based on CES principles and guidelines.
5. **Evaluate:** Examine the partnership by regularly gathering information and obtaining feedback from stakeholders. Evaluate social validity of goals, procedures, and outcomes.
  - Establish and support agreed upon mechanisms for gathering information and feedback from all stakeholders.
  - Make necessary adaptations and adjustments to the collaborative effort in implementing collaborative practices aligned with IPEC and CES principles.
  - Allow opportunities for stakeholders to be involved in documenting, monitoring, and interpreting the success of the effort.
6. **Sustain:** On the basis of what is working, determine if and how to sustain the activities, resources and support, and collaborative partnership(s).
  - Determine how to institutionalize and sustain the CES and interpartner education activities that are working to support the collaborative goals. Determine the stakeholders that need to be involved and in what capacity to sustain the goals.
  - Determine what is needed to maintain the impact, goals, and outcomes of the effort.
  - Commit and be persistent. It will take time to develop and grow the training program. All efforts to build strong community partnerships will not be successful. It requires persistence and commitment to shared values.
7. **Communicate and Celebrate:** Communicate the collective accomplishments, co-learning, and celebrate the collaborative successes.
  - Promote and communicate the goals and outcomes with stakeholders. In collaboration with stakeholders, consider which additional individuals, groups, or audiences should be informed about the work.
  - Highlight the successes and recognize the contributions of all stakeholders, including the client or community.
  - Collaborate in disseminating the work to key audiences and stakeholders. Determine products (e.g., community and scholarly publications, presentations, press releases) that are of value to both the institutional (i.e., academic) and community partners and co-support dissemination activities.

## Case Studies

We offer two case examples  that applied the Participatory Action Cycle for Community Engagement model. They show how an interpartner education and collaboration approach can support training, discovery, application, and integration across stakeholders. Importantly, CES-based collaboration training is not limited to a specific level of education or expertise but can apply to all collaborators involved in the efforts. In the following scenarios, collaborative stakeholders include community partners, as well as both undergraduate and graduate student trainees in behavior analysis programs and adjacent disciplines such as psychology, speech-language pathology, occupational therapy, community health, and education. The case studies demonstrate the implementation of collaborative practices in community settings through interpartner education that aligns with IPEC competencies and CES principles.

### Case Example 1: Undergrads and Underdogs

The first case was co-led by one of the authors, a behavior analyst, and an interdisciplinary colleague, a developmental psychologist. It features the implementation of the Participatory Action Cycle for Community Engagement model through interpartner education. Informally referred to as *Undergrads and Underdogs*, the initiative engaged multiple community partners to address a range of community-identified priorities involving vulnerable populations of children. The complexity of the case reflects both the number of students involved and the diverse funding sources supporting the interpartner education program. While undergraduate engagement was the primary focus, graduate students also contributed significantly, completing thesis and dissertation research as part of the effort.

### Engage and Reflect

Two faculty mentors new to an academic institution prioritized relationship-building with each other and the broader community in support of a shared commitment to advance social and academic wellness among historically underserved populations. To build trust and understand community dynamics, faculty attended events focused on early childhood, education, and literacy, and served on advisory boards and committees, often providing professional development and consultation. These early engagement efforts revealed organizational values and ethics, with shared values viewed as essential for sustainable partnerships. From this foundation, multiple collaborations emerged—some quickly, others after years of relationship-building. While not all contacts led to formal partnerships, ongoing engagement remained critical. Meaningful collaboration required time, humility, and sustained reflection on personal biases, evolving community needs, and institutional capacity.

### Assess

As partnerships formed, faculty dedicated time to listening to community leaders to understand each organization’s mission and unmet needs. Together, they assessed alignment between community goals and their own expertise and institutional capacity. For example, a Head Start program identified a need for adult support during unstructured school hours, prompting faculty to evaluate their ability to contribute meaningfully. This assessment process relied on transparency and open dialogue, including intentional conversations to exchange values, assess interests and capacities, and explore the potential for long-term, mutually beneficial partnerships. The aim was to support community needs while advancing interpartner education and collaboration objectives.

Concurrently, faculty assessed their own capacity and the university’s readiness to support a formal interpartner education program. Two new course designation options, including Service Learning and Global Citizenship, emerged as promising avenues for institutional alignment. Faculty joined related communities of practice and developed a course that met the course designation criteria. The faculty also deepened their understanding of CES principles and IPC (e.g., Kirby et al., 2022; Spencer et al., 2020). Regular meetings with the university’s Office of Community Engagement and Partnerships helped identify existing resources and advocate for infrastructure to sustain and expand the interpartner education model based on CES.

### Plan

Planning with Head Start, the initial community partner, occurred through an iterative and ongoing process of engagement and assessment. Once university mechanisms were established to support collaboration, a replicable process was available to guide future partnerships. However, each initiative required tailored planning based on the partner’s unique context and needs. Planning often took place during site visits or informal meetings, fostering authentic, relationship-centered dialogue. Together, university and community partners co-developed goals, strategies, and addressed logistical considerations such as research protocols, volunteer background checks, and required application approval processes.

Throughout this phase, building trust and cultivating a shared vision remained key priorities. Long-term success depended on reciprocal, mutually beneficial relationships. Two guiding questions anchored planning conversations:

1. Will this partnership help address an unmet community need identified and prioritized by the community?
2. Will this partnership provide a nurturing context for fostering interpartner collaboration skills?

Centering these questions ensured that planning was not merely procedural, but foundational to meaningful, values-aligned collaboration.

### Act

The development of interpartner collaboration skills typically requires sustained engagement beyond a single semester. To address the training, discovery, integration, and application functions of CES, undergraduate students participated in structured collaborative activities over 1.5 to 2 years. A longer-term commitment of students necessitated designing experiences that attracted students across disciplines and maintained continued involvement in the university-community partnerships. Needs identified by community partners influenced the design of the courses and student experiences that would facilitate maintained engagement.

As there were already existing curricular requirements for majors such as behavior analysis, social work, criminology, speech-language science, and public health, curriculum redesign was not feasible. Instead, faculty leveraged the college’s Undergraduate Research Certificate, which required a sequence of research-related courses. Although only a modest number of students pursued the certificate due to limited course offerings, it provided a strategic opportunity to embed interpartner education experiences within the certificate requirements, which was of value to both the students and administrators. The faculty developed three sequential offerings:

1. **Community-Based Research and Intervention (1-credit course)**

Endorsed for both Service Learning and Global Citizenship designations, this course served as an entry point for undergraduate students across disciplines. Offered each fall and spring to small cohorts of ten students, it allowed for close interaction and hands-on engagement. Students committed to three hours weekly at a community site, providing services (e.g., reading to children, delivering interventions) and assisting with research (e.g., data collection).

The course content, including through the readings, assignments, and activities, was designed to develop interpartner collaboration skills and was sequenced around core CES principles: social justice & equity focus, mutual benefit & reciprocity, respect for community knowledge & assets, rigorous & impactful scholarship, and ethical & transparent practices.

Class sessions were discussion-based, with students rotating as facilitators. The faculty modeled facilitation only during the first two sessions and did not lecture. Readings were selected to provoke critical reflection on issues such as race, ethnicity, economics, education, literacy, and cultural humility, which were relevant across all disciplines.

2. **Directed Research (1–3 credits)**

Students who completed the entry course could earn additional credit through Directed Research, which allowed for maintained CES. These individualized experiences enhanced students’ understanding of the scholarship of integration and application, often involving increased time at community sites and direct participation with community stakeholders in opportunities for discovery through research activities.

3. **Research Assistantship (funded)**

Highly engaged students could apply for a competitive assistantship to continue their involvement. These experiences often involved taking on leadership responsibilities and collaborative problem-solving with community partners regarding systems-level implementation challenges.

Undergraduate students participating in Directed Research and Research Assistantships met monthly with faculty mentors and graduate students and were involved in weekly project team meetings. Faculty intentionally modeled and provided feedback to shape positive interpartner interactions, guiding students in reflecting on biases, validating diverse perspectives, asking respectful questions, and appreciating new viewpoints. Undergraduate students worked with different community partners, enabling rich cross-site learning and sharing of similar experiences. Graduate students served as co-mentors, leading weekly project meetings with their undergraduate teams and earning Directed Research credit for their contributions. These interactions deepened collective understanding of CES practices and IPEC competencies.

### Evaluate

Throughout the Undergrads and Underdogs interpartner education program, multiple projects were implemented, each with its own evaluation strategy. The following examples illustrate the range of community collaborations, and the methods used to examine their impact.

**Head Start Partnership.** One of the program’s initial collaborations was with a local Head Start, in which a need for additional adult support during specific times of the day was identified by community stakeholders. The director selected five nearby elementary schools where teachers had expressed this need. Faculty coordinated with teachers to create schedules that aligned with both classroom needs and student availability. Each semester, two undergraduate students were assigned to each classroom, providing 1.5-hour support sessions twice weekly (covering four days of the community-identified need).

Additionally, students were trained to select vocabulary-rich storybooks and design short, embedded vocabulary lessons, which they implemented during classroom visits. Teachers completed end-of-semester surveys evaluating students’ collaboration skills, professionalism, and helpfulness. Feedback was consistently positive, with all teachers expressing interest in continuing the partnership. Undergraduate students also completed reflective surveys on their collaboration experiences, which faculty used to inform ongoing improvements to course design and partner engagement.

**After-School Program Collaboration.** After the Head Start collaboration began, the university’s Office of Community Engagement and Partnerships allocated three work-study positions to the *Undergrads and Underdogs* program. Positive evaluations led to an expansion to 12 positions the following semester, increasing undergraduate student participation. Concurrently, a partnership was formed with the local school district’s after-school program to improve engagement and academic outcomes for primary-grade students in Title I schools. Faculty and program leaders co-designed an initiative in which undergraduates delivered 30-min small-group storytelling and comprehension interventions two to three afternoons per week. The success of this collaboration was highlighted in a short video featured on the university’s website. After-school program leaders completed end-of-year surveys, and students participated in end-of-semester interviews. Feedback from both groups informed ongoing improvements to the initiative.

**School District Research Study.** Building on the success of an after-school initiative, two small districts sought support to implement storytelling interventions in first-grade classrooms. University faculty collaborated with district leaders to design a rigorous study across 10 elementary schools, engaging 32 educators. Graduate students assisted with data collection and undergraduate students coded the data, while a doctoral student in behavior analysis led the project in partnership with 10 school-based speech-language pathologists (SLPs). The project culminated in the doctoral student’s dissertation (Kirby, 2022) and a model implementation study led by a district leader (Staskowski, 2022).

**Children’s Museum Collaboration.** A doctoral student in behavior analysis partnered with a local children’s museum to address the director of education’s need for assessing parent–child engagement across exhibit types. In collaboration with a faculty mentor and the museum director, they co-developed an observation tool to measure interactions such as parent-initiated conversations, duration of engagement, and proximity. Undergraduate students completed the data collection and supported the museum's programming. The museum director and the doctoral student analyzed the data to identify exhibit features—such as manipulatives and tasks requiring adult support—that enhanced engagement.

These diverse evaluation strategies, including surveys, observations, and dissertations, illustrate how the Undergrads and Underdogs initiative integrated community feedback with research rigor to strengthen university-community partnerships and improve interpartner education.

### Sustain

Sustainability was a core goal of the *Undergrads and Underdogs* interpartner education program, with the understanding that community priorities and institutional capacities evolve over time. In some cases, sustainability meant maintaining multi-year partnerships—such as the three-year collaboration with Head Start, which consistently involved the same five teachers until COVID-19 protocols restricted school access. In other cases, sustainability reflected capacity-building with community partners, in which external support became unnecessary over time. For example, the partnership with a local children’s museum led to the co-development and piloting of an observation tool to assess parent–child engagement. Data collected the following semester revealed that exhibits requiring adult support (e.g., writing or helping children with a task) fostered greater parental involvement. On the basis of these findings, the museum refined its design approach, and the director of education now independently uses the tool to evaluate new exhibits. Additional examples (described below) demonstrate both the sustained impact of the interpartner education program and the continued use of interventions developed through CES projects.

**Sustaining the Interpartner Education Program.** The *Undergrads and Underdogs* program has evolved over 15 years, sustained by strong university infrastructure and a broad network of community relationships. When one faculty mentor transitioned to another university, the remaining faculty member maintained key partnerships, projects, and course offerings—demonstrating that the program was not dependent on any single individual. Today, the program remains active, with growing undergraduate participation in CES through the established interpartner education sequence. CES principles and IPEC competencies continue to be embedded throughout the training experiences.

**Sustaining a School-Based Intervention.** In the storytelling and comprehension intervention co-developed with two school districts, 32 educators across 10 schools were initially trained. The partnership has now lasted over eight years, supported by regular collaboration between faculty and district leaders. When a key community liaison retired after six years into the project, their successor sustained the work without interruption. A follow-up survey conducted five years after the original study found that all but one of the trained educators were still using the intervention, with three additional educators having adopted it. These findings highlight the durability of interventions that are co-developed, responsive to community needs, and rooted in strong, mutually beneficial collaboration and stakeholder engagement.

### Communicate and Celebrate

The *Undergrads and Underdogs* CES initiative has generated numerous reasons to celebrate and provided many opportunities for dissemination. While there are too many to list in full, a few key highlights demonstrate the reach and impact of the program.

Across the life of the program:

- Over 20 students completed funded research assistantships.
- Fifteen students were awarded competitive undergraduate research grants.
- Two interdisciplinary teams of undergraduate students published peer-reviewed manuscripts based on their CES projects (e.g., Gardner et al., 2014; O’Reilly et al., 2021).
- Teams held regular celebration events in community sites with refreshments (e.g., lunch, donuts, coffee), involving more stakeholders at various sites, the purpose of which was to share learnings from the collaboration and disseminate plain-language findings.
- The vocabulary lessons and other educator-friendly summaries (e.g., infographics, handouts, videos) are posted on www.TrinasToolbox.com so that educators can download free resources, resulting from the collaborative projects.

Community partners also engaged in knowledge sharing:

- The director of education at the children’s museum authored an article in a professional museum publication, reflecting on the collaboration and the lasting utility of the parent–child engagement observation tool.
- The original district leader from the storytelling and comprehension project was invited to present their experience as an “implementation success story” at a national Implementation Science conference (Staskowski, 2022). This recognition highlighted the power of community–university collaboration and the program’s long-term sustainability.
- Over the years, multiple peer-reviewed manuscripts have emerged from these collaborative projects, co-authored by faculty, students, and community partners.
- More recently, the successor district leader for the storytelling and comprehension project and one of the faculty mentors were invited to speak on an Implementation Science panel, sharing how their sustained collaboration led to long-term impact, meaningful systems change, and eventually the commercialization of the intervention.

These outcomes reflect core CES principles—mutual benefit, community voice, and the dissemination of rigorous, relevant scholarship. Celebration and communication have honored contributors while also strengthening the network of support for future partnerships.

## Case 2: Engaging Stakeholders to Prevent Youth Violence Through Collaborative Practice

The Youth Violence Prevention Center–Kansas City (YVPC-KC), as one of five designated National Centers of Excellence in Youth Violence Prevention, supports an academic–community partnership. Through the YVPC-KC, the ThrYve (Together Helping Reduce Youth Violence) initiative serves as a model of CES, integrating participatory research, co-learning, and community collaboration to address youth violence locally. ThrYve includes multiple components, such as the ThrYve coalition and the implementation of violence prevention programs across community settings—including hospitals, schools, and neighborhoods.

The Participatory Action Cycle was used with community stakeholders to guide the efforts of ThrYve through the YVPC-KC. The online Youth Justice Toolkit (https://youthjustice.ctb.ku.edu/) was developed to support the implementation of the Participatory Action Cycle with the ThrYve coalition and related interpartner initiatives focused on youth justice and prevention (Center for Community Health and Development, 2024). To ensure community accessibility, it is available from the ThrYve website and serves as a guide for implementing the model. Community and academic partners, including youth, families, community residents, and professionals from across systems and fields, participated in structured engagement through collaborative assessment, planning, action, evaluation, and sustainability phases. Although the YVPC-KC is supported through research-related funding, through discovery and integration, there are also applied opportunities that supports the engagement of students and faculty through formalized course training.

### Engage and Reflect

Using a behavioral-community approach, the ThrYve initiative engages a multi-sector coalition of over 40 partners, including both professional and nonprofessional stakeholders (Watson-Thompson et al., 2020). Since 2017, youth and residents have collaborated alongside systems-level partners across more than 15 sectors—such as law enforcement, health agencies, schools, youth-serving organizations, and social service agencies. Faculty, researchers, and students, both graduate and undergraduate, from multiple academic institutions and disciplines have also contributed, including applied behavioral science, public health, social work, family medicine, trauma and acute care surgery, and nonacademic units like admissions, career services, and through research centers.

Organizational structures were established through a coalition and action team structure, which involved regular meetings to support coordination, recognition, and celebration. Five action teams were launched to mobilize change, each with approximately 8–10 partner representatives, including the following: Youth Opportunities, Youth Justice & Crime Prevention, Trauma Support and Social Services, Parent and Community Engagement, and the Youth Advisory Board. Each team included stakeholders whose expertise and lived experience informed strategies such as hospital-based violence prevention, restorative justice programs in schools, crime prevention through environmental design (CPTED) implementation, and expanded out-of-school-time supports.

At the heart of the ThrYve coalition is a commitment to shared ownership, cultural and professional humility, and reflective practice. The coalition established ground rules to guide interactions aligned with IPEC competencies—values and ethics, roles and responsibilities, interprofessional communication, and teams and teamwork. ThrYve supports a range of engagement strategies, bringing together researchers, professionals, students, and community members to co-develop interventions, support after-school programs, facilitate skill-building workshops, and participate in coalition meetings. Rooted in CES principles, these activities model co-learning and reciprocal support toward shared goals.

Reflection is embedded throughout the initiative. Students, instructors, and community partners engage in structured reflection through coursework and fieldwork. Memorandums of understanding support collaboration between students and community partners, often through service-learning designated courses. This reflective practice ensures engagement remains ethically grounded, culturally responsive, and continuously improving.

### Assess

During the assessment phase, the ThrYve coalition convened community partners in coalition meetings, which were initially co-facilitated in partnership with the academic research center and law enforcement officials, to examine youth violence locally. Through the coalition, community stakeholders reviewed information and data alongside the academic partners, which included professors, researchers, and students. In examining the community needs and goals, multiple data review sessions were held with community coalition partners to review and co-interpret local data, issues and concerns surveys, and a root cause analysis with input from youth and adult partners. A youth violence assessment report was developed to guide the collaborative data review process. Additionally, coalition members, including the academic and community partners, were also involved in information gathering processes facilitated by the local health department through the Community Health Assessment, in which the community ranked violence as a top concern.

At the same time, students were engaged throughout the process in ThrYve, including through service-learning courses, independent studies, and practica—particularly within the Applied Behavioral Science program at the University of Kansas (KU). Undergraduate and graduate students were trained in community leadership and core competencies in community health and development, using the Community Tool Box Curriculum (Center for Community Health and Development, 2025).

Through the integration of curricular experiences, students applied their knowledge and were trained through interpartner education and collaboration in the real-world setting, working with partners and youth involved in ThrYve. Through the course students receive instruction in collaboration, partnership development, leadership, and cultural and professional humility. Before participating in ThrYve service-learning activities, all KU students complete the Ethical Service Learning module through the Center for Service Learning. Students involved in research also complete a Community-Engaged Research (CEnR) module, as well as other capacity-building activities including community trainings (e.g., trauma-informed care, ACEs) and technical assistance support (e.g., documentation).

### Plan

The formation of the ThrYve coalition reflects the intentional planning required to build mutually beneficial partnerships through social validity grounded in cultural and professional humility. Early coalition meetings focused on developing a shared vision and mission centered on youth empowerment, safety, and opportunity. Partners collaboratively contributed to identifying the measurable goals to reduce youth violence, including targets such as decreased victimization rates and increased community attachment.

With technical support from the Center for Community Health and Development at the University of Kansas (KU CCHD), the ThrYve coalition facilitated a community action planning process to identify and implement community and systems changes—such as shifts in practice, programming, and policy—to reduce and prevent youth violence. The coalition demonstrates the importance of collaboration in addressing community-level goals. Professionals from 15 sectors work alongside youth (middle school, high school, and college students), families, and residents to co-create and drive change. These 15 sectors include: business, civic organizations, coalitions, community-based organizations, courts and corrections, faith-based groups, health and healthcare, higher education, education, law enforcement, media, social service agencies, state, local, and tribal government, youth-serving organizations, and youth and residents.

By uniting professionals, including practitioners and researchers, with college students, families, and youth under a common mission, the coalition fosters interpartner collaboration and commitment that extends beyond traditional service silos. The coalition organized its work through five action teams, each responsible for selecting and implementing community change strategies. The selected strategies spanned ecological levels—from individual skills training and mentoring to neighborhood-level improvements such as enhanced park lighting and expanded transportation access.

### Act

As the ThrYve coalition entered the implementation phase, action plans developed during the planning process guided the facilitation of selected programs, practices, and policies to address youth violence. In implementing the plan, multiple change strategies were supported by collaborative partners. One example was the development of REVIVE, an evidence-based hospital violence prevention program created and implemented by coalition members. Over several years, REVIVE was developed through interprofessional collaboration among faculty, researchers, and graduate students from multiple departments, in partnership with community stakeholders from the health department, a criminal justice agency, a social service agency, and a local hospital. The program provides support for youth admitted to the hospital due to intentional injury, offering crisis intervention and follow-up services that bridge hospital care with community-based supports. As another example, a youth court strategy was developed with partners from multiple universities in the area, including with involvement from graduate students in applied behavioral science, clinical psychology, and criminal justice programs, in partnership with community partners, including the local district attorney’s office, youth judge, and the local school district.

Through interpartner education, both graduate and undergraduate students engage in practicum and service-learning opportunities alongside professional staff, faculty, and researchers. Undergraduate students work with graduate mentors and program staff to support middle and high school youth participating in the ThrYve after-school program. Additionally, ThrYve supports a youth navigator program which engages high school seniors and recent graduates of ThrYve to provide peer support to fellow ThrYve youth participants while working alongside KU staff and community partners through interpartner education.

A key component of the ThrYve initiative is LEAD UP (leadership, education, and adolescent development with unlimited potential), an academic enrichment initiative that grew through the involvement of service-learning and practicum students in community health and development within the Applied Behavioral Science program at KU. LEAD UP offers college immersion experiences for high school youth participants, paired with tutoring and coaching provided by college students serving as near-peer mentors.

### Evaluate

Evaluation is a core component of the ThrYve initiative and critical for strengthening collaboration, enhancing competencies, and assuring meaningful impact outcomes through continuous reflection, co-learning, and client or community-guided adaptations. The KU CCHD used a web-based data collection and monitoring system that enabled partners and program staff, including trained students, to document activities, monitor strategy implementation, and assess progress toward key objectives. A central evaluation strategy involved documenting community and systems changes, with over 200 changes tracked between 2018 and 2022—demonstrating sustained, cross-sector engagement and implementation of violence prevention strategies. Secondary data—such as youth surveys, police reports, and school outcomes—were reviewed alongside documentation from specific initiatives like REVIVE, the hospital violence prevention program. Through routine monitoring, data sharing, and collaborative review across stakeholders there have been programmatic adaptations and improvements to programs and interventions to enhance effectiveness. Feedback loops are programmed into the approach and regular updates are provided to coalition members and the broader community through monthly meetings, newsletters, presentations, and publications.

**Fig. 4:**
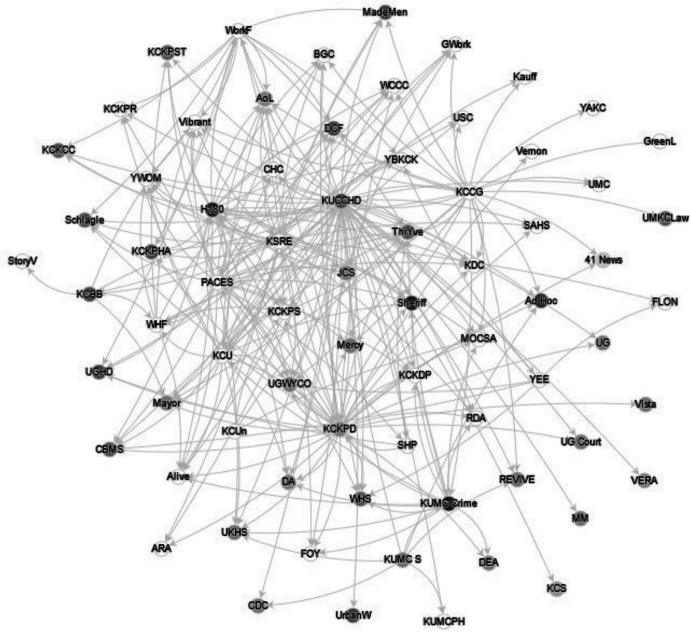
A visual of the network structure of the ThrYve coalition illustrating the relationships, connectivity, and structural dynamics within the coalition to provide insights into partner engagement, collaboration patterns, and potential areas for strengthening inter-organizational ties through a social network analysis (SNA). Each node represents a participating organization, and each line indicates a connection

A graduate student, and co-author of this publication, conducted a student-led social network analysis (SNA) to assess the coalition’s structure, level of collaboration, and changes in engagement patterns over time (Thompson, 2024). SNA provided a behavioral lens for understanding collaborative processes by examining patterns of engagement, trust, and resource sharing across the multi-sector coalition network. By analyzing coalition dynamics, the SNA offered insights into how relationships and structures evolved to support long-term, sustainable systems change. Results from an SNA can inform effective collaborative processes by identifying key coalition connectors, gaps in partner connections, and other factors that may be related to a group’s ability to facilitate meaningful change. For instance, the SNA revealed that partner collaboration spanned Himmelman’s (2002) levels of collaboration, showing evidence of improved connections over time as more partners described their connections as cooperating and collaborating. Additionally, results indicated coalition members remained aligned under a shared vision and trust was sustained over time. On the basis of the initial six years of coalition work, the SNA reaffirmed that decreasing and preventing youth violence remained the coalition’s central focus across collaborative partners (Fig. 4).

### Sustain

Sustainability is a core principle of CES, ensuring that coalition-driven initiatives remain effective and adaptable over time. In addition to direct service delivery, student contributions to sustainability have included collaborating with partners to identify funding opportunities, supporting grant writing, and developing training materials, manuals, and protocols for future student involvement. The ThrYve approach promotes generality, with coalition strategies adopted across multiple contexts to reinforce long-term engagement and impact. For example, youth violence prevention strategies—such as the REVIVE hospital violence prevention program and youth engagement activities—have been integrated into the local community health improvement plan (CHIP), strengthening community ownership and sustainability.

Unlike controlled clinical settings, community-driven approaches require flexibility, as partnerships, funding, and priorities evolve. The success of ThrYve suggests that a well-structured coalition can sustain efforts through shared leadership, strong interpartner collaboration, and embedded community ownership. The ThrYve initiative also illustrates how applied behavior analysis can prepare future professionals to navigate complex collaborative environments, assess coalition dynamics, and contribute to interdisciplinary efforts through interpartner education and collaboration. Many students continue working with ThrYve partners post-graduation, highlighting the role and importance of interpartner education and collaboration supporting training and workforce development related goals and sustainability.

### Communicate and Celebrate

Recognition from both community and institutional stakeholders further legitimizes the initiative and encourages ongoing participation. The accomplishments of the ThrYve coalition and its multistakeholder partners, including youth, college students, and community partners are shared through community presentations, professional conferences, academic publications, and university-hosted student showcases. These venues highlight the success of interdisciplinary, community-engaged partnerships and reinforce the mutual value of academic-community collaboration.

At its core, the ThrYve initiative embodies the principles of CES by fostering participatory, action-oriented approaches to address youth violence. Through use of the Participatory Action Cycle for Community Engagement, the ThrYve initiative and coalition demonstrates how disciplines often partnering to support behavioral-community research and practice, including with applied behavior analysis and public health, can converge to support collaborative practices and sustainable community change. The model serves as a replicable example of how universities can prepare students to navigate complex, collaborative environments and drive transformative impact alongside communities.

## Summary and Implications of Case Studies

The two case examples illustrate the promise and utility of the Participatory Action Cycle for Community Engagement to be applied to the development of CES interpartner education. By aligning with the IPEC competencies, both cases intentionally positioned community stakeholders not as peripheral contributors but as authentic partners—integral to shaping and sustaining meaningful collaborative experiences for students of behavior analysis. These examples showcase how structured, reciprocal partnerships create powerful learning contexts in which behavior analytic students can train, discover, integrate, and apply their scholarship while actively practicing IPEC competencies.

The guidelines for action, based on the case studies, may offer guidance for fostering sustainable, collaborative partnerships. With an eye toward evidence-informed IPE models, the ideas offered here should be considered only a starting place. We are speculating that when behavior analytic students are equipped with a transportable repertoire of collaborative skills—grounded in the principles and values of CES—they are more likely to become humble, trustworthy ambassadors of behavior analytic science and services. This supposition needs to be examined empirically. However, there is good reason to suggest the scalability of our science and practice depends on being welcomed to the table as valued contributors to help develop solutions to societal-level challenges. If disciplinary centrism limits our ability to collaborate effectively with nonbehavioral colleagues and community stakeholders, it must be countered with cultural and professional humility (Kirby et al., 2022).

A key strength of CES models for interpartner education lies in their transformative potential. By decentering disciplinary hierarchies and amplifying community voice, CES can prepare students for interpartner collaboration and elevate the relevance and reach of behavior analytic practices in addressing complex societal challenges. However, this is not yet a standard training model in applied behavior analysis and several barriers may limit the broad implementation of IPECP frameworks. Institutional rigidity, limited faculty experience with CES or IPECP, and variable community readiness can all hinder adoption. Additionally, while CES enhances the depth and authenticity of collaboration, it is inherently time intensive. Trustworthy, reciprocal partnerships require sustained investment, patience, and the willingness to cede power—conditions that may not always align with institutional timelines or incentives. Nonetheless, with intentional design and institutional support, CES interpartner education holds promise for advancing sustainable, community-centered training across diverse settings.

## Conclusion

As the field of behavior analysis reflects on disciplinary centrism and responds to evolving community expectations, interpartner education and collaboration offer a values-aligned path forward. These approaches prepare future behavior analysts to address socially significant problems as reflective, trustworthy partners committed to equity, relevance, and sustained impact.

Interpartner collaboration expands traditional models by including nonprofessional stakeholders—clients, families, and communities—as active contributors alongside professionals. This inclusive framing reflects the applied realities of behavior analytic practice and strengthens the field’s commitment to social validity, cultural responsiveness, and ethical engagement. Embedding this approach within CES creates authentic, reciprocal, and contextually grounded learning experiences. It enables students to engage meaningfully across disciplines and sectors while developing the collaborative repertoire needed to build trust, respond to local priorities, and promote sustainable change.

In contrast to isolated practice or a narrow focus on technical competence, intentional and sustained collaboration fosters person-centered, socially valid work capable of producing lasting, systemic impact. It enhances the discipline’s relevance and credibility, positioning behavior analysts as trusted allies in advancing equitable systems change.

Collaboration also strengthens supervisory and consultative relationships by fostering environments grounded in shared accountability, mutual respect, and open communication. These conditions support ethical decision-making, model best practices, and promote professional growth. While interprofessional education offers one model for advancing collaborative practice, CES provides an alternative framework rooted in integration—across disciplines and sectors—and in training, discovery, and application with and for the communities served.

## References

[CR1] Anderson, L. K. (2023). Autistic experiences of applied behavior analysis. *Autism,**27*(3), 737–750. 10.1177/13623613221118216 35999706 10.1177/13623613221118216

[CR2] Baer, D. M., Wolf, M. M., & Risley, T. R. (1968). Some current dimensions of applied behavior analysis. *Journal of Applied Behavior Analysis,**1*(1), 91–97. 10.1901/jaba.1968.1-91 16795165 10.1901/jaba.1968.1-91 PMC1310980

[CR3] Behavior Analyst Certification Board. (2020). *Ethics code for behavior analysts*. https://bacb.com/wp-content/ethics-code-for-behavior-analysts/

[CR4] Boivin, N., Ruane, J., Quigley, S. P., Harper, J., & Weiss, M. J. (2021). Interdisciplinary collaboration training: An example of a preservice training series. *Behavior Analysis in Practice,**14*(4), 1223–1236. 10.1007/s40617-021-00561-z 34868824 10.1007/s40617-021-00561-z PMC8586302

[CR5] Bowman, K. S., Suarez, V. D., & Weiss, M. J. (2021). Standards for interprofessional collaboration in the treatment of individuals with autism. *Behavior Analysis in Practice,**14*(4), 1191–1208. 10.1007/s40617-021-00560-0 34868822 10.1007/s40617-021-00560-0 PMC8586309

[CR6] Boyer, E. L. (1996). The scholarship of engagement. *Journal of Public Service and Outreach,**1*(1), 11–21. 10.2307/3824459

[CR7] Center for Community Health and Development. (2025). *Action Cycle Model*. University of Kansas. https://ctb.ku.edu/en/get-started

[CR8] Center for Community Health and Development. (2024). *Youth Justice Toolkit.* The University of Kansas. https://youthjustice.ctb.ku.edu/

[CR9] D’Amour, D., Ferrada-Videla, M., San Martin Rodriguez, L., & Beaulieu, M. D. (2005). The conceptual basis for interprofessional collaboration: Core concepts and theoretical frameworks. *Journal of Interprofessional Care,**19*(S1), 116–131. 10.1080/13561820500082529 16096150 10.1080/13561820500082529

[CR10] Fawcett, S., Schultz, J., Watson-Thompson, J., Fox, M., & Bremby, R. (2010). Building multisectoral partnerships for population health and health equity. *Preventing Chronic Disease, 7*(6), A118. https://www.cdc.gov/pcd/issues/2010/nov/10_0079.htm PMC2995607 20950525

[CR11] Friedman, Z. L., El Roy, D., Kerwin, E., Tirri, G., & Broff, A. (2024). Improved soft-skill competencies of ABA professionals following training and coaching: A feasibility study. *Behavior and Social Issues,**33*(1), 64–81. 10.1007/s42822-024-00156-7

[CR12] Gardner, C., Hungate, M., Heitzinger, C., Ewbank, M., & Rogel, L. (2014). Evidence-based practice unites us: A framework for interdisciplinary training and practice. *Journal of Undergraduate Research and Creative Expression,**1*, 1–21.

[CR13] Greenwood, C. R., Bourque, K., Carta, J. J., Walker, D., & Terry, B. (2025). Introduction to the Juniper Gardens Children’s project. *Education and Treatment of Children*. 10.1007/s43494-025-00157-0 41924649 10.1007/s43494-025-00157-0 PMC13037724

[CR14] Harvey, M. T., Harvey, A. C., & McGill, K. L. (2025). Interprofessional collaboration between behavior analysts and mental health professionals. *Behavior and Social Issues*. 10.1007/s42822-025-00219-3

[CR15] Henderson, T. B., Fitzgerald, K. E., Mallory, S. B., & Brodhead, M. T. (2023). The ethical obligations, barriers, and solutions for interprofessional collaboration in the treatment of autistic individuals. *Behavior Analysis in Practice*. 10.1007/s40617-023-00787-z 38076742 10.1007/s40617-023-00787-z PMC10700230

[CR16] Horbanczuk, S., Fettig, A., & Luna, A. (2024). Building collaborative partnerships between behavior analysts and families. *Behavior Analysis in Practice, 17*(4), 996–1007. https://link.springer.com/article/10.1007/s40617-024-00940 10.1007/s40617-024-00940-2 PMC11707203 39790918

[CR17] Himmelman, A. T. (2002). Collaboration defined: A developmental continuum of change strategies. [White Paper]. https://www.myctb.org/wst/CEJ/SiteAssets/CollaborationForAChange.pdf

[CR18] Interprofessional Education Collaborative. (2023). *IPEC core competencies for interprofessional collaborative practice: Version 3*. Interprofessional Education Collaborative.

[CR19] Kirby, M. S. (2022). Oral narrative interventions implemented by teachers, speech-language pathologists, and parents [Doctoral dissertation]. University of South Florida. Retrieved from https://digitalcommons.usf.edu/etd/9390/.

[CR20] Kirby, M. S., Spencer, T. D., & Spiker, S. (2022). Humble behaviorism redux. *Behavior and Social Issues,**31*, 133–158. 10.1007/s42822-022-00092-4 38624848 10.1007/s42822-022-00092-4 PMC8956149

[CR21] Kirkham, P. (2017). ‘The line between intervention and abuse’ – Autism and applied behaviour analysis. *History of the Human Sciences,**30*(2), 107–126. 10.1177/0952695117702571

[CR22] Kelly, A., & Tincani, M. (2013). Collaborative training and practice among applied behavior analysts who support individuals with autism spectrum disorder. *Education and Training in Autism and Developmental Disabilities,**48*(1), 120–131.

[CR23] Lee, J. K., McCutcheon, L. R. M., Fazel, M. T., Cooley, J. H., & Slack, M. K. (2021). Assessment of interprofessional collaborative practices and outcomes in adults with diabetes and hypertension in primary care: A systematic review and meta-analysis. *JAMA Network Open,**4*(2), Article e2036725. 10.1001/jamanetworkopen.2020.36725 33576817 10.1001/jamanetworkopen.2020.36725 PMC7881360

[CR24] O’Reilly, J., Angel, J., Samuel-Lopez, P., Kirby, M., & Spencer, T. D. (2021). Relationship between gender, race, and picture stimulus selection in first graders. *Thrive: Undergraduate Research Journal,**1*(1), 15–21.

[CR25] Pecukonis, E. (2020). Professional centrism and its role in shaping interprofessional education: Implications for social work education. *Journal of Teaching in Social Work,**40*(3), 211–220. 10.1080/08841233.2020.1751776

[CR26] Reeves, S., Perrier, L., Goldman, J., Freeth, D., & Zwarenstein, M. (2013). Interprofessional education: Effects on professional practice and healthcare outcomes. *Cochrane Database of Systematic Reviews,**2013*(3), Article CD002213. 10.1002/14651858.CD002213.pub3 23543515 10.1002/14651858.CD002213.pub3 PMC6513239

[CR27] Reeves, S., Pelone, F., Harrison, R., Goldman, J., & Zwarenstein, M. (2017). Interprofessional collaboration to improve professional practice and healthcare outcomes. *Cochrane Database of Systematic Reviews,**2017*(6), Article CD000072. 10.1002/14651858.CD000072.pub3 10.1002/14651858.CD000072.pub3 PMC6481564 28639262

[CR28] Ruebling, I., Pole, D., Breitbach, A. P., Frager, A., Kettenbach, G., Westhus, N., ... & Carlson, J. (2014). The impact of an interprofessional practice experience on student behaviors related to interprofessional communication and teamwork. *Journal of Interprofessional Care, 28*(4), 299–304. 10.3109/13561820.2014.891571 10.3109/13561820.2013.829421 24000881

[CR29] Slim, L., & Reuter-Yuill, L. M. (2021). A behavior-analytic perspective on interprofessional collaboration. *Behavior Analysis in Practice,**14*(4), 1238–1248. 10.1007/s40617-021-00602-7 34868825 10.1007/s40617-021-00602-7 PMC8586292

[CR30] Sandoval Norton, A. H., & Shkedy, G. (2019). How much compliance is too much compliance: Is long-term ABA therapy abuse*?**Cogent Psychology,**6*(1), Article 1641258. 10.1080/23311908.2019.1641258

[CR31] Spencer, T. D., Slim, L., Cardon, T., & Morgan, L. (2020). *Interprofessional collaborative practice between behavior analysts and speech-language pathologists.* Association for Behavior Analysis International Practice Board. https://www.abainternational.org/constituents/practitioners/interprofessional-collaborative-practice.aspx

[CR32] Staskowski, M. (2022, April). *Implementation of a multitiered oral narrative intervention to boost first graders’ language and literacy skills* [Conference presentation]. Implementation Science in Communication Sciences and Disorders Virtual Conference.

[CR33] Summers, J., Busch, L., Kako, M., & Lau, C. (2022). The role of the behavior analyst on interprofessional mental health teams: Opportunities for collaboration and enhancing patient care. *Journal of Interprofessional Care,**36*(3), 434–440. 10.1080/13561820.2021.1969345 34514941 10.1080/13561820.2021.1969345

[CR34] Thompson, V. (2024). *Examining a community action planning process to mobilize a coalition to prevent youth violence: A case study of the ThrYve Initiative*. ProQuest Dissertations & Theses.

[CR35] U.S. Department of Education, Office of Special Education and Rehabilitative Services. (2022). *Supporting child and student social, emotional, behavioral, and mental health* [Report]. https://www2.ed.gov/documents/students/supporting-child-student-social-emotional-behavioral-mental-health.pdf

[CR36] Virginia Department of Education. (2020). *Guidelines for the provision of applied behavior analysis (ABA) in public schools* [Guidance document]. https://townhall.virginia.gov/L/GetFile.cfm?File=C%3A%2FTownHall%2Fdocroot%2FGuidanceDocs_Proposed%2F201%2FGDoc_DOE_4699_20201215.pdf

[CR37] Watson-Thompson, J., Fawcett, S. B., & Schultz, J. A. (2008). A framework for community mobilization to promote healthy development. *American Journal of Preventive Medicine,**34*(3), S72–S81. 10.1016/j.amepre.2007.12.016 18267205 10.1016/j.amepre.2007.12.016

[CR38] Watson-Thompson, J. (2015). Exploring community engaged scholarship as an intervention to change and improve communities: 2014 recipient of the Ernest A. Lynton Award. *Metropolitan Universities Journal,**26*(1), 11–34.

[CR39] Watson-Thompson, J., Jesson, N., Hassaballa, I., Vanchy, P., Henderson, J., & Moore, C. (2020). Together helping reduce youth violence for equity (ThrYve): Examining the development of a comprehensive multisectoral approach to youth violence prevention. *American Journal of Community Psychology,**66*(3–4), 244–255. 10.1002/ajcp.12449 32865269 10.1002/ajcp.12449

[CR40] Watson-Thompson, J. & Thompson, E. C. (2023). Understanding the importance of intersectionality in advancing community-engaged scholarship and increasing diversity. In R. A. Rehfeldt, T. M. Cihon, & E. B. Rasmussen (Eds.). (2023). *Women in behavior science: Observations on life inside and outside the academy (1st ed.).* Routledge. 10.4324/9781003216773

[CR41] World Health Organization. (2010). *Framework for action on interprofessional education & collaborative practice* (WHO/HRH/HPN/10.3). World Health Organization. Retrieved from https://iris.who.int/bitstream/handle/10665/70185/WHO_HRH_HPN_10.3_eng.pdf?sequence=1

[CR42] Wolf, M. M. (1978). Social validity: The case for subjective measurement or how applied behavior analysis is finding its heart. *Journal of Applied Behavior Analysis,**11*(2), 203–214. 10.1901/jaba.1978.11-203 16795590 10.1901/jaba.1978.11-203 PMC1311293

